# One-Year Clinical Evaluation of the Bonding Effectiveness of a One-Step, Self-Etch Adhesive in Noncarious Cervical Lesion Therapy

**DOI:** 10.1155/2015/984065

**Published:** 2015-02-25

**Authors:** Babacar Faye, Mouhamed Sarr, Khaly Bane, Adjaratou Wakha Aidara, Seydina Ousmane Niang, Abdoul Wakhabe Kane

**Affiliations:** Department of Conservative Dentistry and Endodontics, University of Cheikh Anta Diop-Dakar, BP 5005, Dakar, Senegal

## Abstract

This study evaluated the one-year clinical performance of a one-step, self-etch adhesive (Optibond All-in-One, Kerr, CA, USA) combined with a composite (Herculite XRV Ultra, Kerr Hawe, CA, USA) to restore NCCLs with or without prior acid etching. Restorations performed by the same practitioner were evaluated at baseline and after 3, 6, and 12 months using modified USPHS criteria. At 6 months, the recall rate was 100%. The retention rate was 84.2% for restorations with prior acid etching, but statistically significant differences were observed between baseline and 6 months. Without acid etching, the retention rate was 77%, and no statistically significant difference was noted between 3 and 6 months. Marginal integrity (93.7% with and 87.7% without acid etching) and discoloration (95.3% with and 92.9% without acid etching) were scored as Alpha or Bravo, with better results after acid etching. After one year, the recall rate was 58.06%. Loss of pulp vitality, postoperative sensitivity, or secondary caries were not observed. After one year retention rate was of 90.6% and 76.9% with and without acid conditioning. Optibond All-in-One performs at a satisfactory clinical performance level for restoration of NCCLs after 12 months especially after acid etching.

## 1. Introduction

Currently available adhesives can be categorized into two groups according to the adhesive and clinical application: (i) the etch-and-rinse group, in which a separate etchant is applied and rinsed off, and (ii) the self-etch group, in which an acidic monomer is used to simultaneously demineralize and infiltrate the tooth surface. These adhesives have been simplified and improved to provide better long-term performance [1]. Self-etch adhesives were introduced as an alternative to etch-and-rinse adhesives because of their reduced technique sensitivity and application time [1, 2]. The* in vitro* evaluation of self-etch adhesives by many researchers has led to high values of microtensile bond strength (*μ*TBS), particularly when they are applied to dentin [3–6].

However, a direct correlation between laboratory and clinical results has not yet been shown. Thus, it is difficult to transfer these laboratory results to the clinical setting, where there are many confounding factors. Peumans et al. used class V restorations to clinically evaluate adhesive systems [1]. Noncarious cervical lesions (NCCLs) (including erosion, abrasion, and abfraction) are types of chronic tooth surface destruction that are not bacterial in origin [7, 8]. With increases in the aged population and therefore the number of people who retain their teeth for long periods of time, the prevalence of NCCLs is increasing [8]. The procedures used to treat NCCLs provide excellent opportunities for the evaluation of new materials, because little lesion preparation is needed, and access and restoration are simple. Additionally, the restorations are not load-bearing and thus provide an ideal surface to evaluate the potential of a new adhesive [7]. Furthermore, many clinical studies in conservative dentistry have focused on the treatment of NCCLs because of their increased prevalence and aesthetic implications. Previous results indicated that two-step, self-etch adhesive performs reliably and stably in the clinic [1].

The aim of this study was to evaluate the one-year clinical performance of a one-step, self-etch adhesive system (Optibond All-in-One) combined with Herculite XRV Ultra composite with or without prior etching of the enamel margins in the restoration of NCCLs, using modified United States Public Health Service (USPHS) criteria.

## 2. Materials and Methods

Thirty-one volunteer patients from the operative dentistry clinic at Dakar University (Senegal) were included in this study. The volunteers included 26 males and 5 females who ranged in age from 24 to 73 years. These patients were properly informed about the study, and those who agreed to participate signed a consent form that had been reviewed and approved by the Senegalese National Committee of Ethics. The criteria for inclusion in this study were appropriate oral hygiene, low level of dental decay, absence of periodontal disease, absence of bruxism, and the presence of at least two NCCLs to be restored. All subjects presented a minimum of one pair of NCCLs with a depth greater than or equal to 1 mm.

The NCCLs were restored by the same dental practitioner using self-etch adhesive (Optibond All-in-One, Kerr, Orange, CA, USA) combined with restorative composite (Herculite XRV Ultra, shade A2, Kerr Hawe, Orange, CA, USA). No cavity preparation was performed, and a rubber dam was employed in all cases to prevent contamination.

All patients received symmetrical restorations. On one side, the enamel was etched for 30 seconds with a 36% phosphoric acid gel (De Trey Conditioner 36, Dentsply De Trey, PA, USA). The adhesive was applied in strict accordance with the manufacturer's instructions and light-cured for 20 seconds. The resin composite (Herculite XRV Ultra) was applied using a minimum of two increments and each increment was light-cured for 40 seconds. Any excess composite was removed with a diamond bur, and the restoration was finished with polishing discs (3M Espe, St. Paul, MN, USA). On the opposite side, the same steps were followed without prior etching of the enamel margins.

The practitioner was not involved in the evaluation of the restorations. At 3, 6, and 12 months following restoration, the clinical effectiveness was determined by two qualified evaluators using modified USPHS criteria (Table 1). The evaluators were blinded to the adhesive technique used in any given restoration. Any discrepancy between evaluators was resolved at chair side. Clinical effectiveness was determined in terms of the abovementioned parameters, of which retention (no complete loss of restoration), marginal integrity (severe defects), and clinical microleakage (severe discoloration) were considered as key parameters, determining the overall parameter “clinical success rate.”

The chi-square test was used in the statistical analysis, and *P* < 0.05 was considered to be statistically significant. Statistical analyses were performed using EPI INFO version 6, SPSS 16.0, and Microsoft Excel 2003 software.

## 3. Results

**Figure 1:**
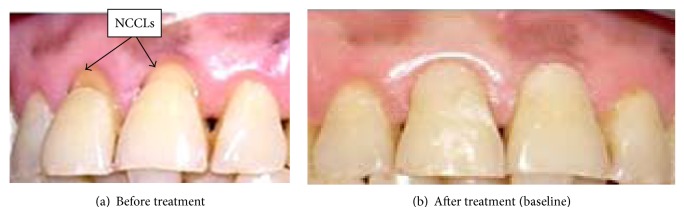
A front view of NCCLs in 11 and 21 before and after restoration.

At baseline, all restorations were 100% successful with regard to the criteria evaluated (retention, marginal integrity, marginal discoloration, and tooth vitality) (Figure 1).

When comparing the criteria at 3 and 6 months, the retention was significantly different only for the restorations performed with prior acid etching of enamel margins (*χ* ^2^ = 4.47; *P* = 0.0344) (Table 2).

At 12 months, 18 out of 31 patients returned for examination, and the recall rate was therefore 58.06%. Out of these patients, 82 teeth were examined, and 100% tooth vitality was recorded for all restorations with or without prior acid etching (Table 2).

After one year retention rate was respectively of 90.6% and 76.9% with and without acid conditioning (Table 3). The performance of restorations at the baseline and after 3, 6, and 12 months of recall time was evaluated by Mc Nemar's test (*α* = 0.05) (Table 4).

## 4. Discussion

The clinical effectiveness of various types of available resin-based adhesives has been reviewed previously [9]. It was concluded that the three-step etch-and-rinse systems provided the most reliable results, and the two-step self-etching systems had good potential [10, 11]. There is a paucity of clinical trial data for All-in-One systems, preventing researchers from making definitive recommendations on these systems [12–14].

In the current study, we evaluated the clinical performance of a one-step, self-etch adhesive combined with a composite in the restoration of 150 NCCLs at baseline and after 3, 6, and 12 months.

Self-etch adhesive systems and composite resins permit restorations with minimally invasive preparation and acceptable aesthetic appearance. Most of the previous studies were conducted* in vitro*, and it is thus difficult to predict the clinical performance of these adhesives and resins. A long-term clinical study will provide the most accurate information regarding the durability of these adhesive restorations, but this type of study will need to span several years with regular recall visits and a high rate of recall for clinical validation. The clinical performance of two-step, self-etch adhesives has been evaluated previously [1]. NCCLs are commonly evaluated for a period of 3 to 5 years [1, 10]. According to Blunck et al., a period of 6 months to 1 year can be sufficient to accurately predict the clinical behavior of an adhesive [15].

The current study evaluated the clinical performance of the Optibond All-in-One one-step, self-etch adhesive system. Of the USPHS criteria, four parameters were utilized to determine the overall clinical success, in accordance with Peumans et al. [9]: retention, marginal integrity, marginal discoloration, and tooth vitality.

Our results at 3 months following restoration showed retention rates of 94.7% and 94.1% with and without prior acid etching of the enamel margins, respectively. The 6-month retention rate was 84.2% for restorations with etched enamel margins and 77% for those without acid etching. After one year, the recall rate was 58.06%. As the missing patients may have lost their restorations, they did not attend the recall appointment. So, this fact may explain low retention rate obtained at 12-month recall. The retention rates were 90.6% and 76.9% with and without acid etching, respectively. This improved performance of restorations with prior acid etching confirmed results reported by Burrow and Tyas [12]. In a one-year clinical trial of the All-in-One adhesive G-Bond, Burrow and Tyas [12] showed that, out of 47 NCCLs etched with phosphoric acid, all restorations were retained (100% retention). However, according to da Costa et al. [16], enamel beveling may not be clinically relevant for the retention of composite restorations in NCCLs after 12 months. Can Say et al. found similar results after one year [17]. In a one-year trial with the same G-Bond adhesive system, Kurokawa et al. [18] also found a retention rate of 100%; however, that study had a small sample size (*n* = 14).

Despite the limited clinical trial data available for All-in-One adhesive systems, the available studies provide promising information with regard to retention. Kubo et al. [14] used one-step self-etch adhesive systems (Clearfil S3 Bond and G-Bond) on 108 NCCLs with beveled enamel margins and ground dentin and found a retention rate of 98.1% after two years. The present study, which did not utilize any enamel or dentin preparations, showed a lower retention rate of 76.9%. Oral hygiene habits may have influenced the durability of the restorations. The majority of the Senegalese population uses sticks for teeth cleaning, which may have affected the retention of our restorations.

Marginal integrity defects (Code C) were observed at 3 months following restoration in 9 teeth (4 with and 5 without acid etching). The preservation of marginal integrity was 94.4% with acid etching and 92.6% without acid etching. Six months after restoration, the rates of marginal integrity preservation were 93.7% and 87.7% with and without acid etching, respectively. At the one-year recall, 94.8% of restorations with prior acid etching showed perfect marginal integrity. Kubo et al. [14] found 100% marginal integrity after 2 years. These findings indicate that marginal integrity in the range of 87.7% to 100% seems to be acceptable with or without enamel preparation.

No marginal discoloration was recorded at 3 months, whereas at the 6-month recall, marginal discoloration was absent in 92.9% of restorations without acid etching and in 95.3% of restorations with etched enamel margins. The results at one year revealed the absence of discoloration for 92.3% of cases with and 80% without acid etching. The one-year results from other clinical trials using the newly developed one-step self-etch systems showed almost no marginal staining [12, 16]. After 2 years, Kubo et al. [14] reported slight marginal staining in 11 restorations using both Clearfil S3 Bond and G-Bond, which represented about 20% of the restorations, while Moretto et al. [19] reported a 1.2% rate of severe marginal defects after 3 years.

A better recall rate (100%) was obtained by Tuncer et al. [20] at 6 and 12 months when using two different microhybrid composites and a two-step etch-and-rinse system in cervical restorations. A satisfactory clinical performance after 12 months was reported after 12 months.

In the present study, we did not observe any loss of tooth vitality. Peumans et al. [1] also described 100% pulp vitality in a study with the two-step self-etch adhesive Clearfil SE.

## 5. Conclusion

Based on modified USPHS criteria, restorations of NCCLs with the one-step, self-etch adhesive Optibond All-in-One in combination with the composite resin Herculite XRV Ultra with prior acid etching of the enamel margins demonstrated clinically acceptable results after one year. Retention was the most critical parameter in the protocol used in this study.

## Figures and Tables

**Table 1:** Modified USPHS rating criteria (de Munck et al.) [5].

Category	Rating	Criteria
Retention	Alpha (A) Bravo (B) Charlie (C)	Retained Partially retained Missing

Marginal integrity	Alpha (A) Bravo (B) Charlie (C)	Closely adapted, no visible crevice Visible crevice, explorer will penetrate Crevice in which dentin is exposed

Marginal discoloration	Alpha (A) Bravo (B) Charlie (C)	No discoloration Superficial staining (without axial penetration) Deep staining (with axial penetration)

Tooth vitality	Alpha (A) Charlie (C)	Present None

Postoperative sensitivity	Alpha (A) Charlie (C)	None Present

Secondary caries	Alpha (A) Charlie (C)	No caries present Caries present

**Table 2:** Three- and six-month evaluations of restorations.

Criteria	Etching	Baseline				3 months				6 months			
		A	B	C	% A + B	A	B	C	% A + B	A	B	C	% A + B
Retention	Yes	76	0	0	100%	72	0	4	94.7%	64	0	8	84.2%
	No	74	0	0	100%	68	0	6	94.1%	57	0	11	77%
Marginal integrity	Yes	76	0	0	100%	68	0	4	94.4%	60	3	1	93.7%
	No	74	0	0	100%	63	0	5	92.6%	50	5	2	87.7%
Marginal discoloration (absence)	Yes	76	0	0	100%	68	0	0	100%	61	1	2	95.3%
	No	74	0	0	100%	62	0	0	100%	53	2	2	92.9%
Tooth vitality	Yes	76	0	0	100%	68	0	0	100%	64	0	0	100%
	No	74	0	0	100%	62	0	0	100%	57	0	0	100%
Postoperative sensitivity	Yes	76	0	0	100%	68	0	0	0%	64	0	0	0%
	No	74	0	0	100%	62	0	0	0%	57	0	0	0%
Secondary caries	Yes	76	0	0	100%	68	0	0	0%	64	0	0	0%
	No	74	0	0	100%	62	0	0	0%	57	0	0	0%

**Table 3:** One-year evaluation of restorations.

Criteria	Etching	Baseline				12 months			
		A	B	C	% A + B	A	B	C	% A + B
Retention	Yes	76	0	0	100%	39	0	4	90.6%
	No	74	0	0	100%	30	0	9	76.9%
Marginal integrity	Yes	76	0	0	100%	31	6	2	94.8%
	No	74	0	0	100%	17	10	3	90%
Marginal discoloration (absence)	Yes	76	0	0	100%	29	7	3	92.3%
	No	74	0	0	100%	19	5	6	80%
Tooth vitality	Yes	76	0	0	100%	39	0	0	100%
	No	74	0	0	100%	30	0	0	100%
Postoperative sensitivity	Yes	76	0	0	100%	39	0	0	100%
	No	74	0	0	100%	30	0	0	100%
Secondary caries	Yes	76	0	0	100%	39	0	0	100%
	No	74	0	0	100%	30	0	0	100%

**Table 4:** Statistical results (Mc Nemar' test).

	Retention	Marginal integrity	Marginal discoloration	Tooth vitality	Postoperative sensitivity	Secondary caries
Chi-square	5.333		5.333			
*P* value	0.021	0.000	0.021	0.093	0.093	0.093
